# High spatial resolution mapping of surface plasmon resonance modes in single and aggregated gold nanoparticles assembled on DNA strands

**DOI:** 10.1186/1556-276X-8-337

**Published:** 2013-07-26

**Authors:** Carlos Diaz-Egea, Wilfried Sigle, Peter A van Aken, Sergio I Molina

**Affiliations:** 1 Instituto de Microscopía Electrónica y Materiales, Departamento de Ciencia de los Materiales e I. M. y Q. I, Facultad de Ciencias, Universidad de Cádiz, Campus Río San Pedro, s/n, 11510, Puerto Real Cádiz, Spain; 2 Max Planck Institute for Intelligent Systems, Stuttgart Center for Electron Microscopy, Heisenbergstraße 3, Stuttgart 70569, Germany

**Keywords:** Plasmonics, Surface plasmon resonance, Gold nanoparticles, Electron energy loss spectroscopy, DNA, Assembly

## Abstract

**Abstract:**

We present the mapping of the full plasmonic mode spectrum for single and aggregated gold nanoparticles linked through DNA strands to a silicon nitride substrate. A comprehensive analysis of the electron energy loss spectroscopy images maps was performed on nanoparticles standing alone, dimers, and clusters of nanoparticles. The experimental results were confirmed by numerical calculations using the Mie theory and Gans-Mie theory for solving Maxwell's equations. Both bright and dark surface plasmon modes have been unveiled.

**PACS:**

78.67.Bf; 61.46.Df; 87.64.Ee

## Background

The field of plasmonics has become a topic of major interest in the last years due to its property of showing an enhancement of the electromagnetic field at a sub-wavelength dimension [1]. This phenomenon is especially noticeable when there is plasmon coupling between metallic nanoparticles that are separated by nanometric gaps [2]. As a result of the overlap of the electromagnetic fields, there are near-field interactions that allow propagation of light [3]. In this effort for designing plasmonic circuits by metal nanoparticle paths, the control of the location of the nanoparticles and the exact separation between them has been achieved, among other procedures, by means of biomolecular nanolithography using deoxyribonucleic acid (DNA) as scaffolds for the gold nanoparticles [4]. With this technique, the inter-particle separation is controlled by the ligand shell allowing angstrom-level precision [5]. To fully characterize such systems, electron energy loss spectroscopy (EELS) has demonstrated to be a very powerful tool since it can probe the local density of states for plasmonic nanoparticles [6], and it has the advantage over optical measurements that it provides information about bright and dark modes.

In this work, we analyze the plasmonic properties of gold nanoparticles attached through DNA strands to a silicon nitride substrate. Individual nanoparticles as well as clusters of them were analyzed by EELS. Spectrum imaging (SI) maps are presented showing dark and bright plasmon modes in these assembled nanoparticles. Analytical calculations based on the Mie theory and Gans-Mie theory for solving Maxwell's equations were performed showing excellent agreement with the experimental results.

## Methods

Energy-filtered transmission electron microscopy and scanning transmission electron microscopy (STEM) EELS SI are two TEM techniques that have been proven to be very powerful when performing plasmonic analysis in small metallic nanoparticles such as silver nanoprisms [7], gold nanoprisms [8], silver nanorods [9], and nanowire dimers [10]. Both techniques present advantages and disadvantages [11]. The intensity of the LSPR peaks for small nanoparticles (the ones analyzed here have diameters between 5 and 25 nm) is very low, making EELS in STEM the best choice allowing both, very high spatial resolution and fine sampling of the energy loss spectrum.

For the work presented here, the SI maps were acquired using the Zeiss sub-electronvolt-sub-angstrom-microscope operated at 200 kV. This equipment is located at the Stuttgart Center for Electron Microscopy (Stuttgart, Germany). It is equipped with a Schottky field emitter, an electrostatic monochromator, and the high-dispersion and high-transmissivity in-column MANDOLINE filter [12]. The spectrometer dispersion was set to 0.01377 eV per channel for the 2,048 channels with an exposure time of 0.2 s per spectrum. The spatial sampling used was in the range of 1.9 to about 2.6 nm per pixel giving a total acquisition time of between 10 and 20 min for every single SI. The energy resolution achieved, measured as the full width at half maximum of the zero loss peak, was between 138 and 151 meV. Before and after the SI acquisition, high-angle annular dark-field (HAADF) images were taken in the selected area to control spatial drift.

Using the peak at zero energy loss, the SI is realigned in energy to correct energy shifts from one pixel to the other. To mitigate the noise in the spectra, principal component analysis (PCA) was used to decompose the entire map and reconstruct it without the very high-order components [13]. The zero loss peak (ZLP) removal was performed using a power-law function. For every localized surface plasmon resonance (LSPR) peak, one Gaussian function was fitted to the curve by nonlinear least squares fit algorithm. The energy loss maps and the amplitude maps were created using the center of the fitted Gaussian function and its amplitude, respectively.

For the case of a single nanoparticle standing alone, theoretical calculations were done to support the results. The calculations were performed using routines based on the MATLAB toolbox MNPBEM [14]. To estimate the LSPR response of one gold nanosphere, the Mie theory was used to solve the Maxwell equations using both the quasistatic approximation and solving the full Maxwell equations. In that way, the light extinction of such a sphere was used to match the energy loss results acquired at the microscope. In the same way, for the case of one single-standing nanoellipsoid, the Mie-Gans theory was used using the quasistatic approximation.

To fabricate the samples used for this work, DNA strands were deposited on a silicon nitride grid surface. These DNA strands were used as biomolecular templates for the self-assembly of gold nanoparticles [4]. These samples were acquired from Dune Sciences (Eugene, OR, USA). The fabrication process was described elsewhere, and it is not included here because this process is not the aim of this work.

## Results and discussion

**Figure 1:**
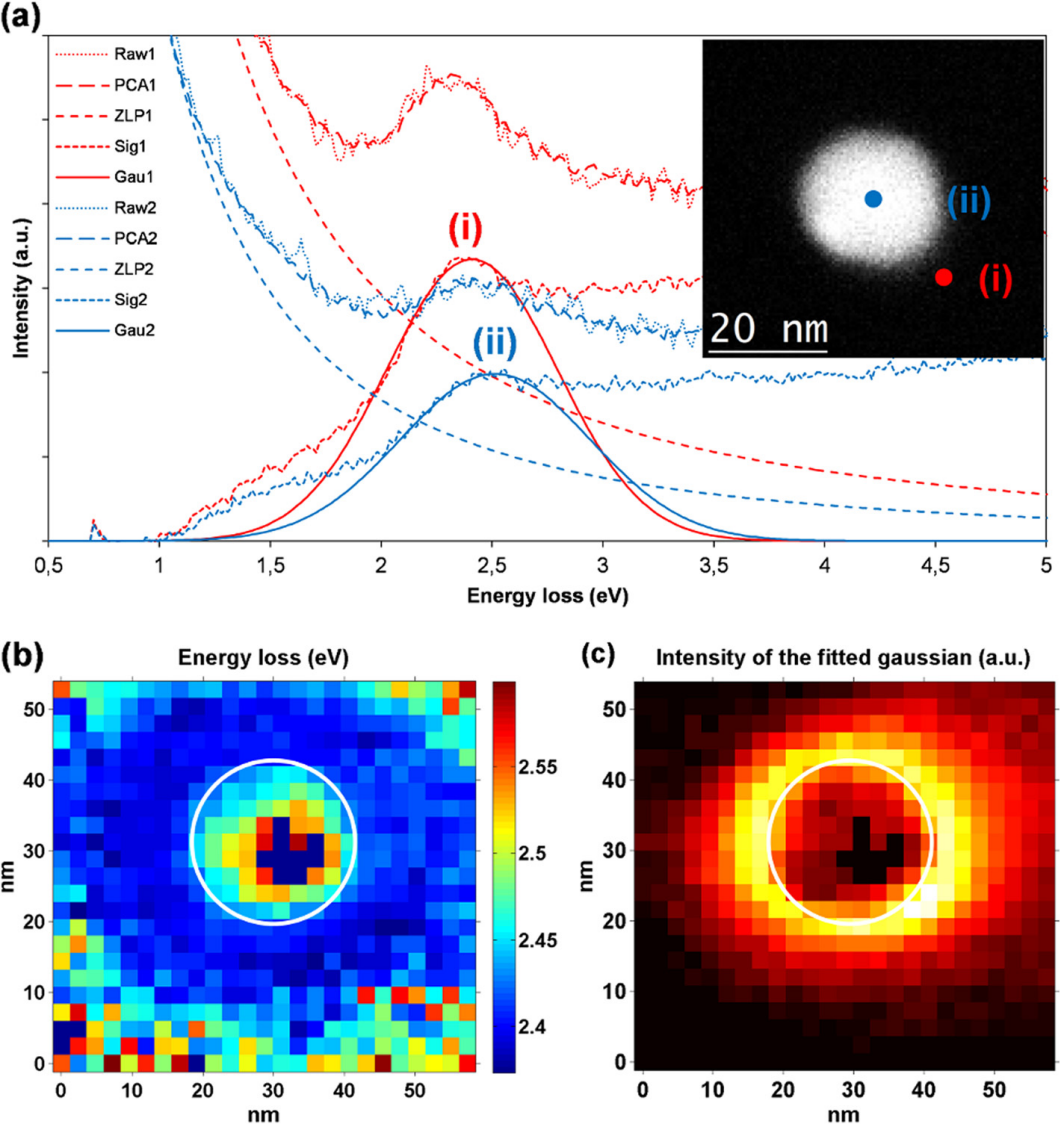
**Electron energy loss spectra (a) and energy loss (b) and amplitude (c) maps. (a)** Electron energy loss spectra of a 26-nm gold nanosphere linked through DNA strands to a Si_3_N_4_ membrane; the inset shows an HAADF image of the nanoparticle. The spectrum marked as (curve i) shows the energy loss along the trajectory marked with a red dot where a resonance peak can be clearly seen at 2.4 eV, the one marked as (curve ii) shows the peak at 2.5 eV approximately corresponding to the trajectory through the nanoparticle marked with the blue dot. **(b)** Energy loss map displaying the value of the center of the fitted Gaussian to the LSPR peak. **(c)** Amplitude map with the intensity value of the center of the fitted Gaussian to the LSPR peak.

Figure 1 shows the results of the LSPR analysis performed on a 26-nm gold spherical nanoparticle linked through DNA strands to a silicon nitride membrane. The top-right corner inset in (a) shows a high-angle annular dark-field (HAADF) image of the area where the SI was acquired including the gold spherical nanoparticle. Two representative EELS spectra marked by the two colored dots are displayed in the chart. The raw data extracted from the SI are displayed using dotted lines. After applying PCA, the results are shown using dashed lines with long dashes. The result after ZLP subtraction is shown as dashed lines with medium-sized dashes. The difference between the data after PCA reconstruction and the ZLP fit is displayed in the chart using dashed lines with small dashes. The Gaussian fit function is shown with solid lines. Energy loss and amplitude maps are shown in Figure 1b,c. The chart in (b) uses a color-scale that goes from blue as the lowest energy value to red as the highest one. The chart in (c) uses a color-scale that ranges from black, through red and yellow to white as the highest amplitude value for the fitted Gaussian.

Both the energy map and the spectrum labeled in red as (curve i) show a very distinct peak at 2.4 eV, this is the typical value for a dipolar LSPR mode in a gold nanoparticle of this size in air [15,16]. To validate the results, the Mie theory has been used to solve the Maxwell equations using both the quasistatic approximation and solving the full Maxwell equations. A 26-nm gold sphere standing in vacuum was considered yielding both approximations a result of 2.44 eV for the extinction of light with the absorption as the main contribution over scattering which corresponds for a metal nanoparticle of this size [1]. The influence of the silicon nitride substrate explains the slight blueshift of the resonance peak.

The bulk plasmon resonance can also be seen in the energy map showing values between 2.45 and 2.55 eV. One of these spectra marked with the blue dot and labeled as (cuve ii) is shown for display. It clearly shows a resonance peak at 2.5 eV, that resonance peak is broader and less intense than that of the LSPR. Similar results have recently been reported for silver nanoparticles with comparable sizes [17].

**Figure 2:**
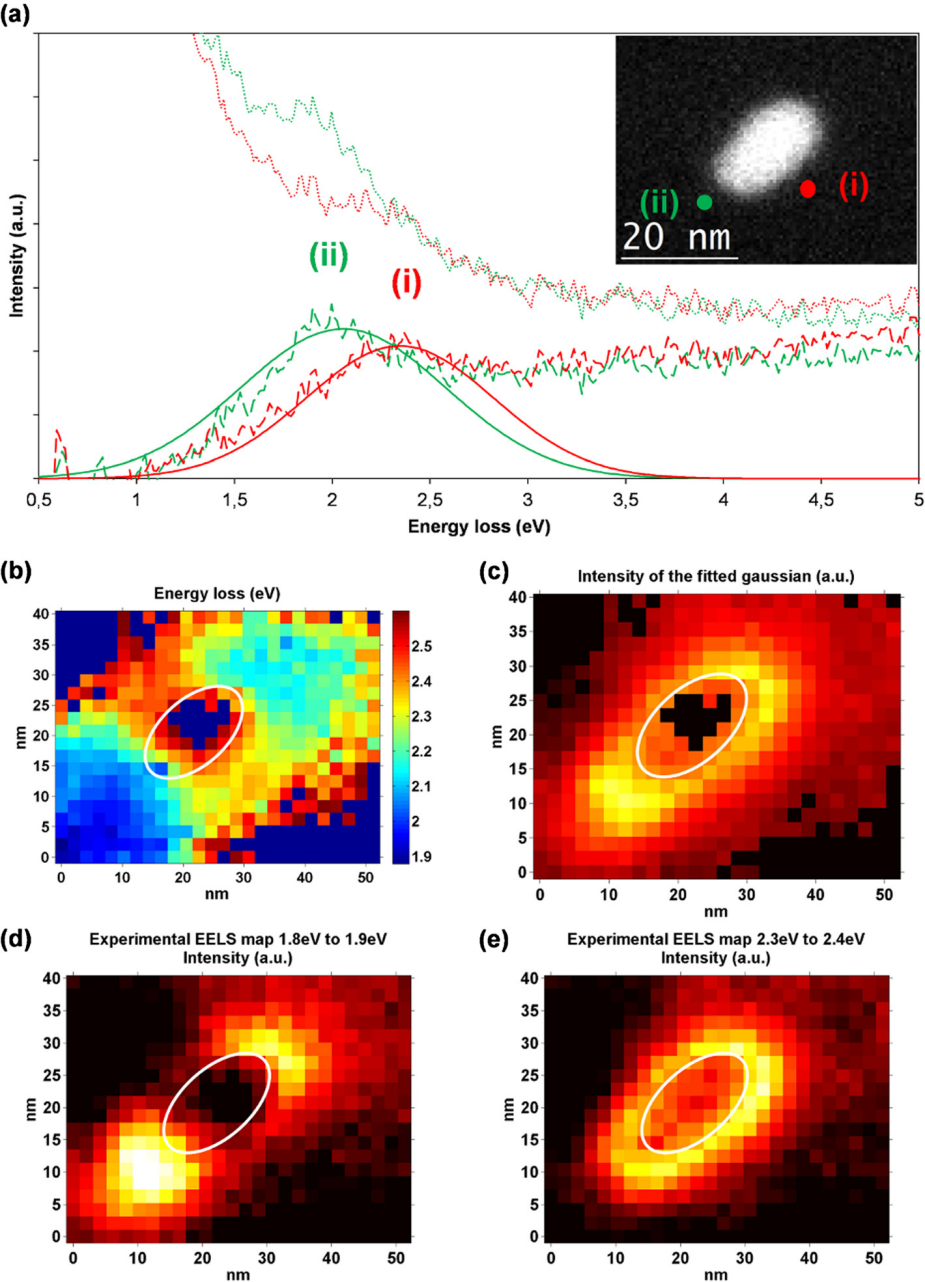
**Electron energy loss spectra (a) and energy (b), amplitude (c), and energy-filtered (d,e) maps. (a)** Electron energy loss spectra of a 21-nm × 11-nm gold nanoellipsoid linked through DNA strands to a silicon nitride membrane. The inset shows an HAADF image of the nanoparticle. Two representative spectra have been selected and displayed, the first one shown in red (curve i) has a resonant peak at 2.4 eV corresponding to the typical dipolar mode, and the peak of the second one in green (curve ii) is at a lower energy value, 2.15 eV. The two modes can also be identified in the energy map **(b)** that presents the values of the centers of the fitted Gaussian to the LSPR peak. The amplitude map with the value of the center of the fitted Gaussian to the LSPR peak is shown in **(c)**. The charts in **(d)** and **(e)** show the energy-filtered maps centered in the abovementioned modes.

The results of the LSPR analysis on a gold ellipsoidal nanoparticle are shown in Figure 2. The nanoparticle-long axis measures 21 nm while the short one is 11-nm long. The chart in (a) displays two illustrative EELS spectra that were acquired in the positions marked by colored dots in the top-right corner inset that shows an HAADF image of the area where the SI was acquired including the gold ellipsoidal nanoparticle. The graph shows, in dotted lines, the raw data extracted from the SI, in dashed lines, the difference between the data after PCA reconstruction and the ZLP fit, and in solid lines, the fitted Gaussian functions. Two modes are clearly identifiable, (curves i and ii). Both of them are dipolar bright modes, the mode labeled as (curve i) is located at 2.4 eV, and it is usually named transversal mode since it induces a dipole perpendicular to the long axis of the ellipsoid when excited with transversal polarization. A second mode can clearly be seen at 2.15 eV, it has been labeled as (curve ii). This is usually called a longitudinal mode, the exciting electron beam, when located near the ends of the long axis of the ellipsoid induces a dipole along that long axis that is red-shifted with respect to the transversal mode due to the longer distance. In the energy map (b), the light blue and dark blue areas correspond to the low-energy (curve i) mode, while the yellow and orange zone marks the area where mode (cuve ii) dominates. The mode identified as (cuve i) shows a higher intensity with respect to mode (curve ii), this can be seen in chart (c). To further illustrate the analysis, graphs (d) and (e) show energy-filtered maps for the values of the dominant modes. These maps were created by removing the ZLP in the same way as before and then integrating the signal within an energy interval, namely 1.8 to 1.9 and 2.3 to 2.4 eV, respectively.

The HAADF image reveals that the nanoparticle is not perfectly symmetrical. There is intensity decay along the long axis of the nanoparticle from top to bottom indicating a higher volume of gold on the top part of the nanoparticle. Profiles of the nanoparticle perpendicular to the longitudinal axis also reveal that this one is slightly thicker on the top and a little bit sharper at the bottom. This shape is confirmed by the energy and intensity maps where an asymmetry can be seen between top and bottom of the nanoparticle. The energy at the top corresponds to 2.15 eV, while at the bottom, a red shift down to 2.1 eV and below is visible. However, the main characteristic of the sharper part of a nanoparticle is that it presents a higher intensity of the field, this can be seen in both the intensity map (c) and the energy-filtered map (d).

Similar to the sphere calculations, the Mie-Gans theory was used to validate the findings using the quasistatic approximation for non-spherical particles. An ellipsoid was modeled estimating its axis to be 21, 11, and 11 nm. It was assumed to be surrounded by vacuum. Two modes for extinction of light at 2.47 and 2.33 eV are found. Both modes seem to be red-shifted with respect to the experimental results which are possibly attributable to the effect of the substrate.

**Figure 3:**
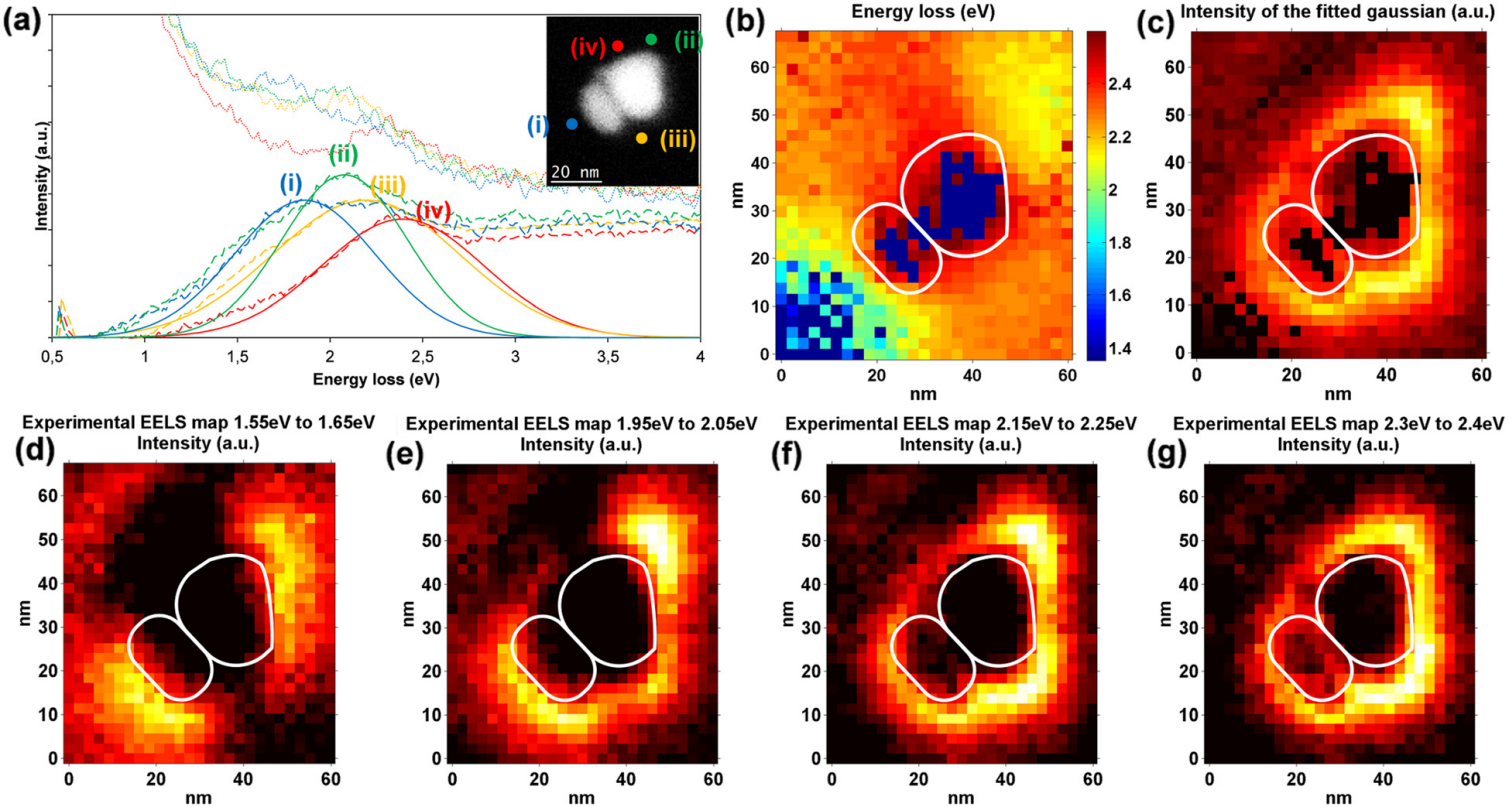
**Electron energy loss spectra (a) and energy (b), intensity (c), and energy-filtered (d,e,f,g) maps. (a)** Electron energy loss spectra of a dimer of gold nanoparticles linked through DNA strands to a silicon nitride membrane for the trajectories denoted on the HAADF image of the inset. The resonance peaks for (curves i, ii, iii, and iv) are located at 1.9, 2.1, 2.3, and 2.4 eV, respectively. **(b)** Energy map of the centers of the fitted Gaussian to the LSPR peaks. **(c)** Amplitude map with the value of the center of the fitted Gaussian to the LSPR peak. **(d,e,f,g)** Energy-filtered maps centered at 1.6, 2.0, 2.2, and 2.35 eV.

Figure 3 shows the outcome of the LSPR analysis of two linked gold nanoparticles. The top-right corner inset in (a) shows an HAADF image of the area where the SI was acquired. Both nanoparticles can be seen there. The top-right one measures 27 nm × 22 nm, while the bottom-left one is 23 nm × 12 nm in size. Together, they form a dimer of 35 nm × 27 nm, approximately. Complex modes are exposed and at least four different zones can be distinguished. One EELS spectrum has been extracted for each of these areas, and it is represented in (a) with different colors. In the same way as before, the dotted lines in the graph correspond to the raw data extracted from the SI, the dashed lines to the difference between the data after PCA reconstruction and the ZLP fit, and the solid lines show the fitted Gaussian functions. The energy map (b) and intensity map (c) are also presented. The lowest energy area is well represented by the spectrum (curve i) which corresponds to the light blue zone in the energy map. This is a rather intense zone with energy values near 1.9 eV. The spectrum shown in green (curve ii) exemplifies the yellow area in the top right part of the dimer with the highest intensity values and energies close to 2.1 eV. Spectrum (curve iii) is also from a very high intensity zone with energy values near 2.3 eV, as marked by the orange colors in the energy map. Finally, the highest energy mode is located in the red area of the energy map at 2.4 eV as it can be seen in spectrum (curve iv). Graphs (d, e, f, and g) show energy-filtered maps created by integrating the signal without ZLP within an energy interval of 0.1 eV around the energies 1.6, 2.0, 2.2, and 2.35 eV.

One way to explain the depicted modes is to assume the dimer as a big nanoparticle of 35 nm × 27 nm. One such nanoparticle would behave in the same way as the one analyzed in Figure 2 with a low-energy mode along the long axis and a high-energy one perpendicular to it. The former would correspond to the areas marked as (curves i and ii) and the last to the areas labeled as (curves iii and iv). The symmetry of each of these two global modes is broken by the irregular shapes of the individual nanoparticles.

**Figure 4:**
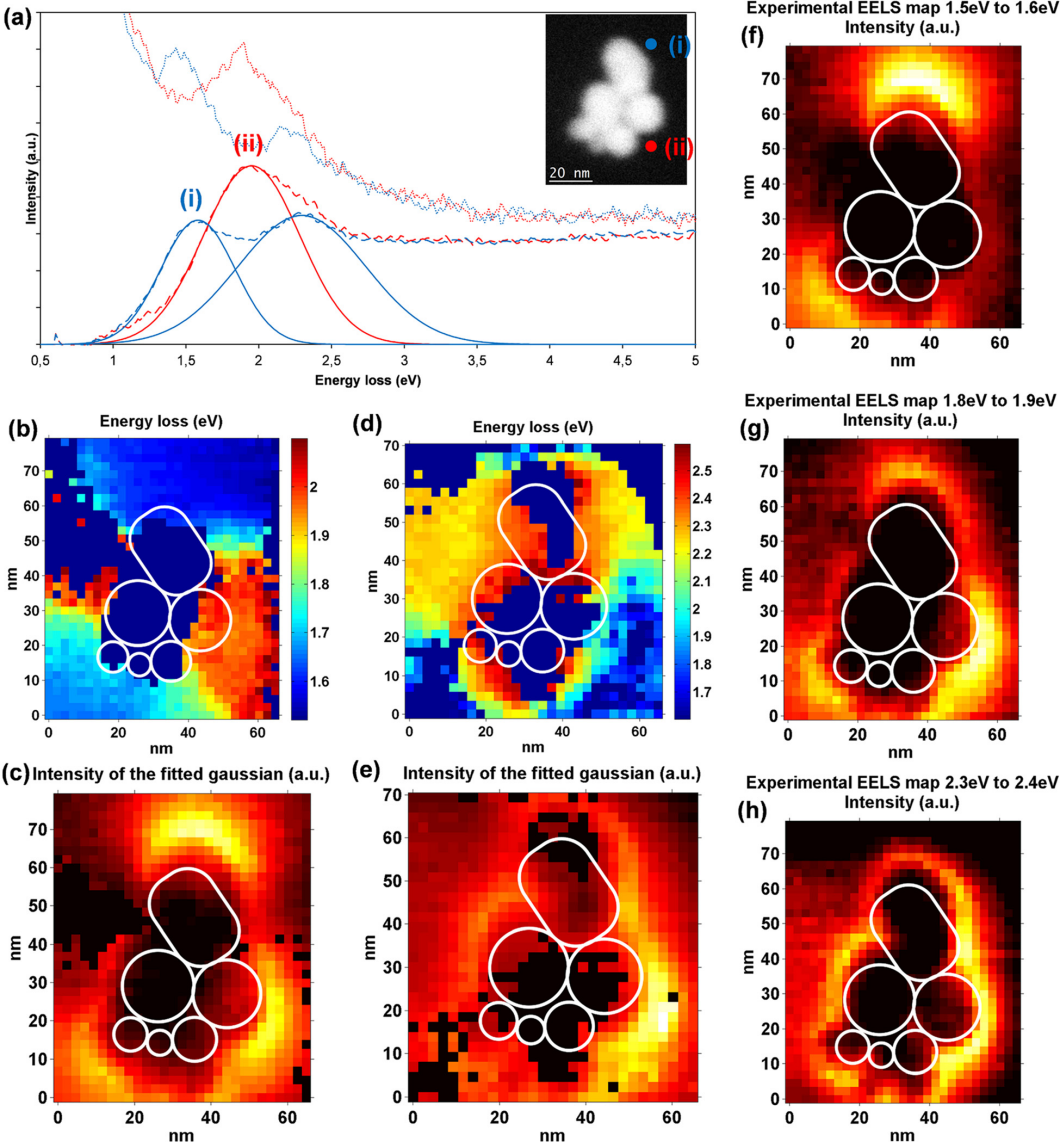
**Electron energy loss spectra (a), energy (b,d), amplitude (c,e) energy-filtered (f,g,h) maps. (a)** Electron energy loss spectra of a cluster of gold nanoparticles linked through DNA strands to a silicon nitride membrane for the trajectories indicated on the HAADF image of the inset. For the first trajectory (curve i), two resonance peaks can be seen at 1.6 and 2.3 eV; for the trajectory (curve ii), there is a strong LSPR at 1.9 eV. To better illustrate the two resonant modes on the trajectory (curve i), two energy maps **(b,d)** are presented with the centers of the fitted Gaussian to the LSPR peaks. The amplitude of the fitted Gaussian can be seen in the amplitude maps **(c,e)**. The energy-filtered maps centered at 1.55, 1.85, and 2.35 eV are presented in **(f,g,h)**.

A bigger cluster formed by six gold nanoparticles is shown in Figure 4. Two representative spectra are shown in (a) with an HAADF image of the area where the SI was acquired in the inset. The aggregate of nanoparticles includes one ellipsoidal nanoparticle of 29 nm × 20 nm and five almost spherical ones with the following diameters: 20, 19, 16, 12, and 9 nm. Two EELS spectra are shown in (a) with red and blue lines, respectively. The raw data are shown using dotted lines, the curve after PCA and ZLP subtraction is shown in dashed lines and the fitted Gaussian functions in solid lines. Two energy maps are displayed, each of them covering different energy values. The one shown in (b) displays the value of the center of the fitted Gaussian for those ones located between 1.5 and 2.1 eV, while (c) represents the amplitude of that function in every point. The energy map (d) was built with the energy values between 1.8 and 2.6 eV. The intensity map (e) shows the amplitudes of the fitted Gaussians. The reason for splitting the energy map into two energy regions is that there is an area where two modes dominate with similar intensity. The charts labeled as (f, g, h) are energy-filtered maps created by integrating the signal without ZLP within the energy intervals 1.5 to 1.6, 1.8 to 1.9, and 2.3 to 2.4 eV, respectively.

In order to describe the plasmonic behavior of the structure, three main areas are highlighted. The most intense surface plasmon mode is located in the bottom-right corner of the map. It is represented by the red spectrum (curve ii), the orange area in (b), and the light blue zone in (c). The energy values for this area are close to 1.9 eV. There is a second plasmonic mode of about 1.6 eV which is located both at the top and at the bottom of the cluster. It is displayed using blue colors in the energy map (b), and it corresponds to the lowest energy curve in the blue line (curve i) shown in (a). There is a third area with energy values of 2.3 eV that is located at the upper part of the cluster at its left and at its right. It can be identified with the yellow and orange colors in the map labeled (d). This mode coexists with the lowest energy one in the area selected with (curve i), and that is why it is shown as the highest energy value curve in the spectrum (a). It can be seen in both the intensity map (e) and the energy filtered map (h) that, although this mode is more intense at the right side of the structure, it also exists almost symmetrically at the left side.

In the same way as we did for the dimer of nanoparticles, we can consider the cluster to be a big nanoparticle with a vertical long axis and a horizontal short one. The area in the extreme of the long axis would be the one with blue colors in Figure 4b. This area is again the one with the lowest energy while the high-energy area is the one with orange colors in the same figure. However, for such a complex cluster, these two modes are significantly modified by the irregular shapes of the individual nanoparticles creating areas with two modes like the one depicted by (curve i). It is remarkable that the areas with the highest intensity for the resonance peaks are the sharper areas of the cluster.

## Conclusions

In conclusion, the plasmonic properties of gold nanoparticles assembled on DNA strands were investigated at nanometer scale by EELS. This analysis was done for isolated nanoparticles, dimers, and clusters. The results were compared to analytical calculations showing good agreement. It was shown that the LSPR peak appears at 2.44 eV (508 nm) which is typical for isolated spherical gold nanoparticles. For an elongated particle, two modes are identified, a longitudinal and a transversal mode. A dimer of nanoparticles was analyzed with the result of complex modes being exposed. The dimer as a whole seems to present longitudinal and transversal modes behaving similarly to an elongated nanoparticle with the size and shape of the dimer but with the modes shifted by the irregular shapes of the individual nanoparticles. A cluster of six nanoparticles was analyzed with similar results. The use of EELS unveiled bright and dark plasmon modes. The low-energy ones are located on the extremes of the long axis and the high-energy ones on the short axis. The sharper areas of the cluster present higher intensity in the resonance peak. The results presented in this manuscript contribute to the design of plasmonic circuits by metal nanoparticle paths.

## Abbreviations

DNA: Deoxyribonucleic acid; EELS: Electron energy loss spectroscopy; TEM: Transmission electron microscopy; STEM: Scanning transmission electron microscopy; SI: Spectrum imaging; HAADF: High-angle annular dark-field; PCA: Principal component analysis; ZLP: Zero loss peak; LSPR: Localized surface plasmon resonance.

## Competing interests

The authors declare that they have no competing interests.

## Authors’ contributions

CDE has designed the study, participated in the acquisition of the EELS maps, and carried out the alignment and reconstruction of the data; he has taken part in discussions and in the interpretation of the result and has written the manuscript. WS has participated in the design of the study, acquired the EELS maps, taken part in discussions and in the interpretation of the result, and revised the manuscript. PAvA has supervised the research and revised the manuscript. SIM has conceived the study, participated in its design, and supervised the manuscript and the experimental part. All the authors have read and approved the final manuscript.

## Authors’ information

CDE is a Ph. D. student at the Universidad de Cádiz. WS is a Research scientist at the Stuttgart Center for Electron Microscopy (StEM), Max Plank Institute for intelligent systems, PAvA is head of the Stuttgart Center for Electron Microscopy (StEM), Max Planck Institute for intelligent systems. SIM is a full professor at the Departamento de Ciencia de los Materiales e Ingeniería Metalúrgica y Química Inorgánica, Universidad de Cádiz.
